# P01.42. Dose-dependent effects of massage-like loading in an animal model following eccentric exercise

**DOI:** 10.1186/1472-6882-12-S1-P42

**Published:** 2012-06-12

**Authors:** T Best, Y Zhao, D Jarjoura, X Zhang, C Haas

**Affiliations:** 1 The Ohio State University, Columbus, USA

## Purpose

Determine the effects of duration, magnitude, and frequency of massage-like compressive loading on the recovery of active muscle properties (torque - joint angle) following a bout of damaging eccentric exercise.

## Methods

Twenty-four New Zealand white rabbits were surgically instrumented and underwent a bout of damaging eccentric exercise to the tibialis anterior muscle. Rabbits were randomly assigned to a protocol with massage frequency of 0.25 or 0.50Hz at a compressive force of 5 or 10N for 15 or 30 minutes. The contralateral limb served as the exercised, non-massaged control. A torque-angle relationship was obtained for 21 tibiotarsal joint angles, pre- and post-exercise, and post four consecutive days of massage (applied by customized device). Peak isometric torque was the primary outcome variable. Muscle wet weight and histological analysis were also performed at the end of the protocol.

## Results

Greatest recovery of peak isometric torque occured at the 0.5Hz, 10N, 15min condition showing a recovery index of 1.083 [RI]. Analysis showed a significant difference for the RI of the massaged hindlimb between the two magnitudes (5 and 10 N; p=0.004) and the two frequencies (0.25 and 0.5Hz; p=0.012) but no difference for the two durations (15min and 30min). Muscle wet weight showed massaged animals tissue weighing (n=12) 3.22 +/- 0.61g, while the control (non-massaged) tissue weighed (n=9) 3.74 +/- 0.65g (p<0.05). Histology verified the beneficial effects of massage through decreased myofiber damage and cellular infiltration.

## Conclusion

There is a dose-response effect with respect to magnitude and frequency of massage-like compressive forces on recovery of active muscle properties following eccentric exercise in an animal model. These results may help to explain the variability in human studies evaluating the efficacy of this therapy for recovery from intense exercise.

